# Identification of the intersegmental plane by arterial ligation method during thoracoscopic segmentectomy

**DOI:** 10.1186/s13019-022-02011-5

**Published:** 2022-11-04

**Authors:** Haiqi He, Heng Zhao, Lei Ma, Kun Fan, Jinteng Feng, Rui Zhao, Xiaopeng Wen, Jia Zhang, Qifei Wu, Junke Fu, Guangjian Zhang

**Affiliations:** 1 Department of Thoracic Surgery, The First Affiliated Hospital of Xi’an Jiaotong University, Xi’an, 710061 China; 2 Department of Surgery, The First Affiliated Hospital of Xi’an Jiaotong University, Xi’an, 710061 China

**Keywords:** Segmentectomy, Intersegmental plane, Thoracoscopy

## Abstract

**Background:**

Thoracoscopic segmentectomy is a common surgical procedure in thoracic surgery today. However, identifying the intersegmental plane is difficult in the surgical process. Therefore, we evaluated the feasibility of the arterial ligation method for determining the intersegmental plane and compared the demarcation status with the intravenous indocyanine green (ICG).

**Methods:**

We retrospectively reviewed the records of 35 patients with peripheral small lung nodules who underwent thoracoscopic segmentectomy between May and December 2020. First, the preoperative three-dimensional reconstruction was performed to distinguish the location of lung nodules and the anatomical structures of targeted segmental arteries, veins, and bronchi. Second, the targeted segmental arteries were ligated, and the intersegmental plane was determined by the inflation-deflation technique. The waiting time for the appearance of the inflation-deflation line was recorded. Thirdly, the intersegmental plane was identified again using the ICG fluorescence method. Finally, the consistency of the two intersegmental planes was evaluated.

**Results:**

The intersegmental planes were successfully observed in all patients using the arterial ligation method. Thirty-four patients underwent segmentectomy as planned, and one patient finally underwent lobectomy due to insufficient surgical margin. The waiting time for the appearance of the intersegmental plane by arterial ligation method was 13.7 ± 3.2 min (6–19 min). The intersegmental planes determined by the arterial ligation method and the ICG fluorescence method were comparable, with a maximum distance of no more than 5 mm between the two planes. The mean operative duration was 119.1 ± 34.9 min, and the mean blood loss was 76.9 ± 70.3 ml. No evident air leakage was found during the operation. Only one patient experienced a prolonged air leak (≥ 5 days) during the postoperative recovery. No atelectasis occurred in all cases. The chest tube duration was 3.1 ± 0.9 days.

**Conclusion:**

The arterial ligation method can efficiently and accurately identify the intersegmental plane, comparable to the ICG fluorescence method.

## Introduction

With the popularization of low-dose computed tomography (CT), more and more early-stage lung cancers, especially those with the image manifestations of ground-glass nodules (GGNs), are being detected [1, 2]. Thoracoscopic segmentectomy has become the primary treatment method for early-stage non-small cell lung cancer (NSCLC). Compared with lobectomy, segmentectomy can achieve comparable oncological outcomes in early-stage lung cancer while preserving more normal lung tissue [3–5]. Segmentectomy is preferred with wedge resection because of its more adequate surgical margin and superior oncological outcomes [6].

Segmentectomy, however, is a more complicated surgical procedure than wedge resection or lobectomy. Determining the intersegmental plane is one of the difficulties in thoracoscopic anatomic segmentectomy. The most common way to identify the intersegmental plane is the inflation-deflation method, which is achieved by inflating residual segments, leaving the targeted segment atelectatic, or vice versa [7]. However, this method may lead to the inaccurate intersegmental plane due to the presence of Kohn pores, Lambert canals, and direct airway anastomosis, and sometimes it is challenging to identify the intersegmental plane in patients with emphysema. Another way to determine the intersegmental plane is the vascular method, which can be achieved by near-infrared fluorescence imaging with intravenous ICG or ligation of the targeted segmental artery [8, 9]. The ICG fluorescence method is more accurate but technically more complicated. As a simpler approach, closing the targeted segmental artery alone had been shown to determine the intersegmental plane [9]. However, few studies evaluated the efficiency and accuracy of arterial ligation in determining the intersegmental plane [10]. To this end, we compared the arterial ligation method with the ICG fluorescence method in identifying the intersegmental plane.

## Methods

### Patients

We retrospectively reviewed the records of 35 patients who underwent thoracoscopic anatomic segmentectomy in our department between May and December 2020. The criterion for inclusion was patients planned to undergo thoracoscopic anatomical segmentectomy for peripheral small lung nodules. The exclusion criteria were allergy to iodine. All patients underwent high-resolution chest CT with sections of 1-mm thickness before the surgery. Three-dimensional (3D) reconstruction of bronchi, arteries, and veins was performed using Mimics 22.0 software (Materialise, Belgium). This study was approved by the Ethics Committee of The First Affiliated Hospital of Xi 'an Jiaotong University. Written informed consent was obtained from each patient before surgery.

### Operative procedure

**Fig. 1:**
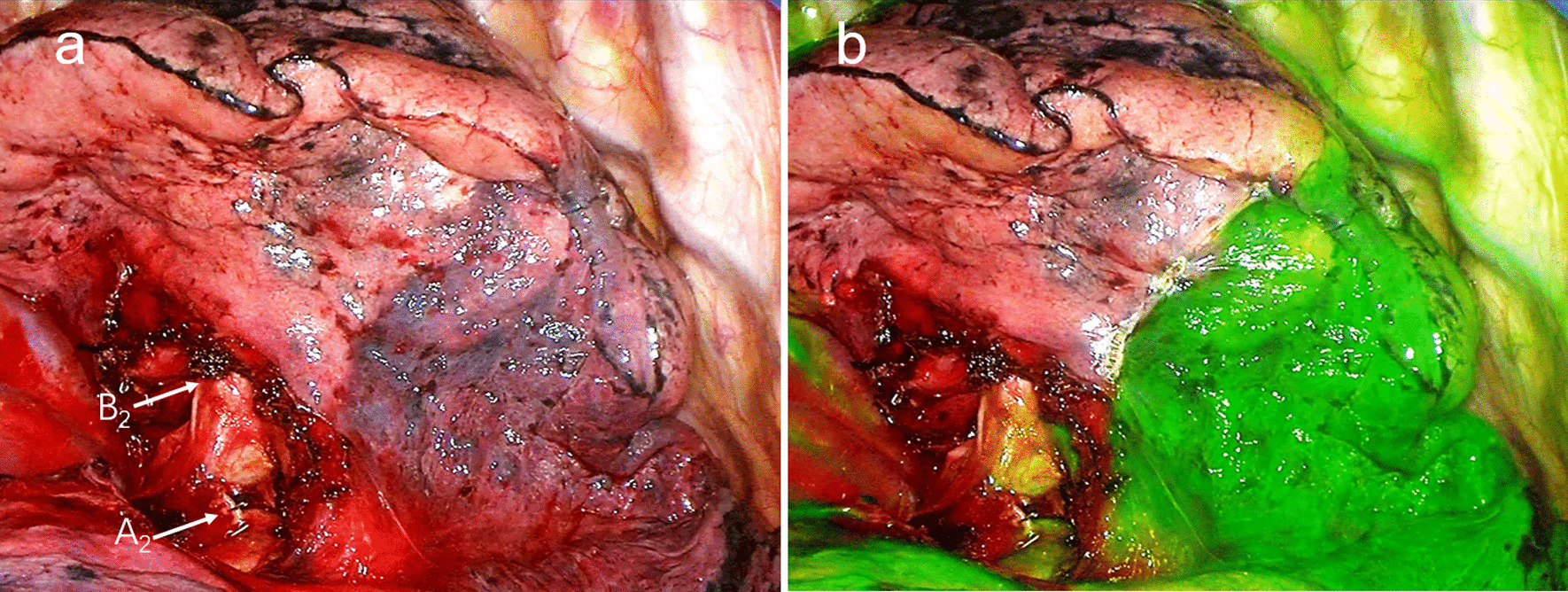
Identifying the intersegmental plane during the right S2 segmentectomy. **a** The first intersegmental plane was identified by the arterial ligation method. The targeted segmental artery (A^2^) and bronchi (B^2^) were dissected and the artery was ligated. **b** The second intersegmental plane was identified by the ICG fluorescence method. The marking line on the visceral pleura was the first intersegmental plane, and the boundary of the green fluorescence was the second intersegmental plane

All patients were intubated with a double-lumen tube and placed in a lateral decubitus position. A 12-mm camera trocar was placed in the seventh or the eighth intercostal space on the midaxillary line. A 3-cm operation hole was made in the fourth or fifth intercostal space on the anterior axillary line. First, Under the guidance of preoperative 3-D reconstruction (Fig. 1), the targeted segmental arteries, veins, and bronchi were distinguished, and the segmental arteries were ligated. Then, bilateral pulmonary ventilation was performed with pure oxygen to fully inflate the entire pulmonary lobes. After that, contralateral unilateral lung ventilation was performed again, and the surgical procedures were stopped at this point. While waiting, the surface of the lung was observed through the thoracoscopy until the intersegmental plane was clearly presented. The time from the contralateral unilateral ventilation to the appearance of the intersegmental plane was recorded. Then, the intersegmental plane was marked on the visceral pleura using electrocautery. Next, the second intersegmental plane was observed by an infrared thoracoscopy system (HyPixel™ R1, Shenzhen Mindray Bio-Medical Electronics Co., China) after intravenous injection of 3-ml indocyanine green (ICG) solution (ICG solution was prepared by dissolving 25 mg ICG in 10 ml distilled water). The intersegmental line was still marked using an electrocoagulation hook. The maximum distance between the two marking lines was used to assess the consistency of the two intersegmental planes. When the distance was greater than 5 mm, the two intersegmental planes were considered to be inconsistent. At this time, the resection of the targeted segment was performed according to the intersegmental plane determined by the ICG fluorescence method. For the division of lung parenchyma, the central part was dissected along the intersegmental veins with an electrocoagulation hook or ultrasonic scalpel. The peripheral part was divided along the visceral pleura marking using the stapler. After segmentectomy, a test of air leakage from the lung was implemented by inflating the lung underwater.

## Results

A total of 35 patients met the inclusion criteria. Patient characteristics are shown in Table 1. The lesions were located in the right upper lobe (14 cases, 40.0%), right lower lobe (6 cases, 17.1%), left upper lobe (11 cases, 31.4%), and the left lower lobe (4 cases, 11.4%). The intersegmental planes were successfully identified in all patients by the arterial ligation method and ICG fluorescence method in turn (Fig. 1). Finally, 34 patients underwent planned thoracoscopic segmentectomy. Only one patient underwent lobectomy immediately after segmentectomy because of the insufficient surgical margin. The resected segments were as follows: right lung: S^1^ (5 cases), S^2^ (4 cases), S^2^ + S^1a^ (2 cases), S^3^ (3 cases), S^6^ (3 cases), S^8^ (3 cases); left lung: S^1+2^ (2 cases), S^3^ (1 case), S^1+2^ + S^3^ (4 cases), S^3b+c^ (1 case), S^4^ + S^5^ (3 cases), S^6^ (1 case), S^9^ (1 case), S^10^ (1 case), S^9^ + S^10^ (1 case). The mean operative time was 119.1 ± 34.9 min. The mean operative blood loss was 76.9 ± 70.3 ml. The pathological diagnoses are shown in Table 1, and none of the lymph nodes were metastatic.

**Table 1:** Characteristics and postoperative outcomes of patients

Characteristics	Value
Age (years, mean ± SD)	56±11
Gender (n (%))	
Male	7 (20%)
Female	28 (80%)
Pulmonary function	
FEV_1_ (L)	2.00±0.18
Lesion location (n (%))	
Right upper lobe	14 (40%)
Right lower lobe	6 (17%)
Left upper lobe	11 (31%)
Left lower lobe	4 (11%)
Pathological diagnosis (n (%))	
Benign	2 (6%)
Atypical adenomatous hyperplasia	3 (9%)
Adenocarcinoma in situ	10 (29%)
Minimally invasive adenocarcinoma	9 (26%)
Invasive adenocarcinoma	11 (31%)
Surgical margin	
≥2 cm or the diameter of the tumor	34
<2 cm or the diameter of the tumor	1
Operative time (min)	119.1±34.9
Intraoperative blood loss (mL)	76.9±70.3
Duration of chest drainage (days)	3.1±0.9
Postoperative complications, n (%)	
Prolonged air leaks (> 5 days)	1
Atelectasis	0

*FEV1* forced expiratory volume in 1 second.

For the arterial-ligation method, the time spent waiting for the appearance of the intersegmental plane was 13.7 ± 3.2 min (6–19 min). This time was 13 s (10–15 s) for the ICG fluorescence method. The consistency of the two intersegmental planes was evaluated by the distance between the two marking lines. The maximum distance was found to be no more than 5 mm. Thus, the intersegmental planes determined by the two methods were consistent (Fig. 1), and we believe that the intersegmental plane identified by the arterial ligation method was accurate.

No evident air leakage was found during the operation. Only one patient experienced a prolonged air leak (≥ 5 days) during the postoperative recovery. No atelectasis occurred in all cases. The chest tube duration was 3.1 ± 0.9 days (2–6 days).

## Discussion

Pulmonary lobectomy is the standard surgical procedure for early-stage lung cancer, further supported in 1995 by the Lung Cancer Study Group’s randomized trial [11]. Recently, several studies have demonstrated that segmentectomy can achieve equivalent short-term surgical results and long-term oncological outcomes for patients with early-stage NSCLC compared to lobectomy [4, 5, 12]. Furthermore, compared with lobectomy, segmentectomy offers the advantage of preserving whole lung function [13, 14]. Therefore, segmentectomy is not only a reasonable choice for patients with poor lung function but also a curative surgery for patients with stage IA lung cancer. However, segmentectomy is considered technically challenging, preventing its widespread application.

Determining the intersegmental plane is one of the most critical steps of segmentectomy. Inaccurate identification of the intersegmental plane may lead to excessive resection of the parenchyma, insufficient surgical margin, and even residue of the lesions. This condition can also increase postoperative complications, such as air leakage, atelectasis, and hemoptysis. Therefore, several techniques have been proposed to identify the intersegmental plane accurately [8, 15–20].

The conventional inflation-deflation technique identifies the intersegmental plane by inflating the target segment and deflating the preserved segment, with the disadvantage of limiting the thoracic operating space. Therefore, the modified inflation-deflation technique was more commonly used [15]. However, the inflation-deflation line may be unclear and inaccurate because of the collateral ventilation via the Kohn pores, the Lambert canals, and the direct airway anastomosis [7]. Alternatively, the resected segment inflation technique can be used to determine intersegmental planes, which selectively inflates the target segment through bronchial jet ventilation [16], air injection into the target bronchus using a butterfly needle [17], or bronchial ligation with a slip knot before whole lung deflation [18]. Due to collateral ventilation, these methods share the same potential limitation of inaccurate intersegmental planes. The staining technique by bronchial injection of dyes, such as methylene blue or ICG, has also been used to identify intersegmental planes [19, 20]. This method can not only make the intersegmental line appear on the lung surface but also make the lung parenchyma of the target segment stained. However, a potential problem with this approach is that the dye can spread into adjacent segments through the Kohn pores.

The near-infrared fluorescence mapping with intravenous ICG has become an innovative technique, which can provide a clear view of the segment borders [8, 21]. This method is based on differential blood flow between the resected segment and the residual segment caused by the severing of the target segmental artery. The advantage of the ICG fluorescence method is that it does not require intraoperative lung re-inflation and thereby doesn’t affect maneuver space during video-assisted thoracic surgery. Additionally, another diffusion-based method is the arterial-ligation technique proposed by Iwata et al. [9], in which only the pulmonary artery was ligated before lung inflation, without ligation of the bronchus and the vein. Recently, Fu et al. [10] showed that the arterial-ligation method is feasible and effective in identifying the intersegmental plan compared to the modified inflation-deflation method. To further validate the feasibility and effectiveness, we compared the arterial ligation method with the ICG fluorescence method. Our results suggested that the intersegmental plane determined by the arterial ligation method is comparable to the ICG fluorescence method. Moreover, we found that this approach is helpful for subsegmental resection and combined subsegmental resections, as shown in others [9, 10, 22]. Notably, the arterial ligation method is much more convenient than those above. However, it takes a longer time to wait for the appearance of the intersegmental plane compared to the ICG fluorescence method. The major problem with the arterial ligation method is difficulty determining the intersegmental plane in patients with severe emphysema [10]. In our study, the intersegmental planes were successfully identified in all patients, as none of the included patients had severe emphysema.

The mechanism for identifying the intersegmental plane using this method was explained by Iwata et al. [9] from the perspective of gas exchange. The oxygen in the alveoli of the resected segment cannot enter the bloodstream through the gas exchange because of arterial ligation. In contrast, the oxygen in the residual segments can be taken away by blood flow. Thus, an inflation-deflation line appears between the resected segment and residual segments. Based on this theoretical basis, we can speculate that severing both the arteries and veins of the target segment would be more favorable for the appearance of the inflation-deflation line. In the study by Fu and colleagues [10], it was shown that the waiting time for the arterial ligation method was a little longer than that for the inflation-deflation technique. However, this mechanism has not been experimentally confirmed. If this is true, the waiting time for the appearance of the intersegmental plane will vary when the lung is inflated with different gases, which needs to be verified in future studies.

In conclusion, the arterial ligation method can accurately identify the intersegmental plane during the segmentectomy, a simple and effective alternative to other methods. However, the underlying mechanism remains to be further experimentally confirmed.

## Data Availability

All patients in this study were from the First Affiliated Hospital of Xi 'an Jiaotong University. Informed consent was signed by patients before the operation. All procedures performed in this study involving human participants are in accordance with the ethical standards and the 1964 Declaration of Helsinki and its later amendments or comparable ethical standards.
